# Draft Genome Sequences of Two *Pseudoalteromonas porphyrae* Strains Isolated from Seagrass Sediment

**DOI:** 10.1128/genomeA.00092-16

**Published:** 2016-03-17

**Authors:** Ruth D. Lee, Guillaume Jospin, Jenna M. Lang, Jonathan A. Eisen, David A. Coil

**Affiliations:** a University of California Davis Genome Center, Davis, California, USA; b University of California Davis, Department of Evolution and Ecology, Department of Medical Microbiology and Immunology, Davis, California, USA

## Abstract

Here, we present the draft genome sequences of *Pseudoalteromonas porphyrae* UCD-SED9 and UCD-SED14 (phylum *Proteobacteria*). These strains were isolated from sediment surrounding the roots of the seagrass, *Zostera marina*, collected near the UC, Davis Bodega Marine Laboratory (Bodega Bay, California). The assemblies contain 4,847,456 bp and 4,817,752 bp, respectively.

## GENOME ANNOUNCEMENT

We aimed to culture organisms as part of the seagrass microbiome project (http://seagrassmicrobiome.org/), a collaboration between researchers at the University of California, Davis and the University of Oregon. This project focused on assessing the microbial communities living on and around common eelgrass (*Zostera marina*). Morphologically distinct microorganisms, able to grow on a diverse set of media, were selected for sequencing. In the presented paper, both *Pseudoalteromonas porphyrae* UCD-SED9 and UCD-SED14 strains were isolated from sediment surrounding *Zostera marina* roots near the University of California, Davis Bodega Marine Laboratory (Bodega Bay, California, USA). The sampling site was located north of Westshore Park, California (38°19′10.0″N, 123°03′13.8″W).

*Pseudoalteromonas porphyrae* is a marine bacterium, previously found to be associated with both marine and land plant growth-promotion (1). More specifically, some of *P. porphyrae*’s oxidoreducing enzymes have been associated with an increase in stress tolerance in these plants, promoting their growth and development (1).

1:100 and 1:1000 dilutions of sediment in “seawater media” (15.0 g of agar, 5.0 g of peptone, 2.0 g of beef extract, 0.5 g of KNO_3_, and 1.0 liters of InstantOcean) were made and spread on seawater media plates, grown at room temperature for 24 h, and individual colonies were double dilution streaked. A Wizard genomic DNA purification kit (Promega) was used to extract DNA from fresh 5-mL seawater media overnight cultures.

A Nextera DNA sample prep kit (Illumina) was used to make paired-end libraries (Illumina). Libraries were sequenced on an Illumina MiSeq, at a read length of 300 bp. A total of 1,711,239 and 1,289,818 high-quality paired-end reads were processed by the A5-miseq assembly pipeline for strains UCD-SED9 and UCD-SED14, respectively (2, 3). This pipeline automates quality control, error correction, contig assembly, and data cleaning. The resulting assembly consisted of 97 contigs for UCD-SED9 (longest: 621,678 bp; *N*_50_: 183,595) and 117 contigs for UCD-SED14 (longest: 469,157; *N*_50_: 146,422); these contigs were submitted to GenBank. The final assembly of UCD-SED9 contained 4,847,456 bp with a G+C content of 39.7% and had an overall coverage estimate of ~177×. The final assembly of UCD-SED10 contained 4,817,752 bp with a G+C content of 39.8% and had an overall coverage estimate of ~134×. Genome completeness was assessed using the PhyloSift software (4), which searches for a list of 37 highly conserved, single-copy marker genes (5), of which all 37 were found in both assemblies.

The Rapid Annotations using Subsystems Technology (RAST) server was used to perform automated annotations on both strains (6–8). The *Pseudoalteromonas porphyrae* UCD-SED9 and UCD-SED14 assemblies contain 4,308 and 4,303 predicted protein-coding sequences, respectively and 133 and 141 predicted noncoding RNAs, respectively. The full-length 16S rRNA sequences were obtained from the RAST annotation and used for identification of the isolates. Examination of BLAST search results and phylogenetic analyses (9) identified both strains as *Pseudoalteromonas porphyrae*.

### Nucleotide sequence accession numbers.

Both whole-genome shotgun projects have been deposited at DDBJ/EMBL/GenBank under the accession numbers LITL00000000 (for UCD-SED9) and LHPH00000000 (for UCD-SED14). The versions described in this paper are LITL00000000.1 (for UCD-SED9) and LHPH00000000.1 (for UCD-SED14).
